# Intraoperative angioedema induced by angiotensin II receptor blocker: a case report

**DOI:** 10.1186/s13037-018-0174-0

**Published:** 2018-09-20

**Authors:** Ala”a Alhowary, Haitham Odat, Obada Alali, Ali Al-Omari

**Affiliations:** 1 Department of Anesthesiology and critical care, King Abdullah University Hospital, Ar Ramtha, Jordan; 2 Faculty of Medicine, Jordan University of Science and Technology, P.O.Box: 953, Irbid, 21110 Jordan; 3 Division of Otolaryngology, Department of Special Surgery, King Abdullah University Hospital, Ar Ramtha, Jordan; 4 Division of orthopedics, Department of Special Surgery, King Abdullah University Hospital, Ar Ramtha, Jordan

**Keywords:** Angioedema, Intraoperative complications, Angiotensin receptor antagonists

## Abstract

**Background:**

Angiotensin II receptor blockers are a class of antihypertensive agent that is developed to exclude the adverse effects of angiotensin converting enzyme inhibitors. However, as angiotensin II receptor blockers have begun to be more widely prescribed, cases of angiotensin II receptor blocker-induced angioedema have been reported. Rare cases of angioedema following surgery in patients using angiotensin converting enzyme inhibitors have been published.

**Case presentation:**

A 38-year-old man with past history of hypertension was admitted for an elective lumbosacral spine surgery. He had been taking Valsartan 160 mg a day for the past 4 years.

At the end of the surgical procedure and turning the patient into supine position, we noticed severe swelling in the neck and the face with.an edematous tongue, floor of the mouth, glottis, and supraglottic areas. A diagnosis of drug induced angioedema was made and intravenous dexamethasone, diphenhydramine and ranitidine were given. The patient remained intubated and was transferred to the intensive care unit. The valsartan was suspected to be the precipitating factor for the angioedema and was therefore discontinued.

The swelling started to regress after 2 h, and resolved completely by the third day.

**Conclusion:**

The precise mechanism of angiotensin II receptor blocker-induced angioedema is still unknown and should be thoroughly investigated. This report demonstrates a unique case of intraoperative angiotensin II receptor blocker-induced angioedema. Potential differential diagnoses of postoperative facial edema are discussed in detail, including the prolonged prone positioning for posterior spine surgery. Anesthesiologists should be aware of such rare, but potentially dangerous, perioperative adverse reaction that can occur with angiotensin II receptor blockers use.

## Background

Angioedema is a non-pitting edema that occurs in the face, neck and mucous membranes. It is a potentially life-threatening condition because it may lead to upper airway obstruction [1].

Antihypertensive angiotensin converting enzyme (ACE) inhibitors are considered as one of the precipitating factors for angioedema [2].

Angiotensin II receptor blockers are generation of antihypertensive drugs which are developed to avoid the side effects of ACE inhibitors like cough and angioedema. However, reported cases of angiotensin II receptor blocker-induced angioedema have been discussed in literature [3].

Few cases of angioedema following anesthesia in patients using ACE inhibitors have been reported. [4–7]. However to the best of our knowledge, none has reported intraoperative angiotensin II receptor blocker-induced angioedema.

Herein we present a case of intraoperative angioedema induced by valsartan in a hypertensive patient during lumbosacral spinal fusion surgery.

## Case presentation

A 38-year-old man with past history of hypertension was admitted for a lumbosacral spine surgery. He had been taking Valsartan 160 mg a day for the past 4 years. He underwent two uneventful previous surgeries before diagnosis of hypertension and was not known to have prior drug intolerance or atopy with unremarkable family history. He had no history of nonsteroidal anti-inflammatory drugs in the perioperative period.

The patient was admitted for an elective spinal fusion surgery at L5–S1. His vital signs, airway examination, other physical examination, and laboratory tests were unremarkable. On the next day, the patient was taken to the operating room; anesthesia was induced with intravenous fentanyl and propofol, smooth tracheal intubation was done using atracurium. The patient was turned to prone position and anesthesia was maintained with isoflorane and fentanyl. The patient was given 10 mg morphine and 1 g cefazolin intraoperatively.

**Fig. 1:**
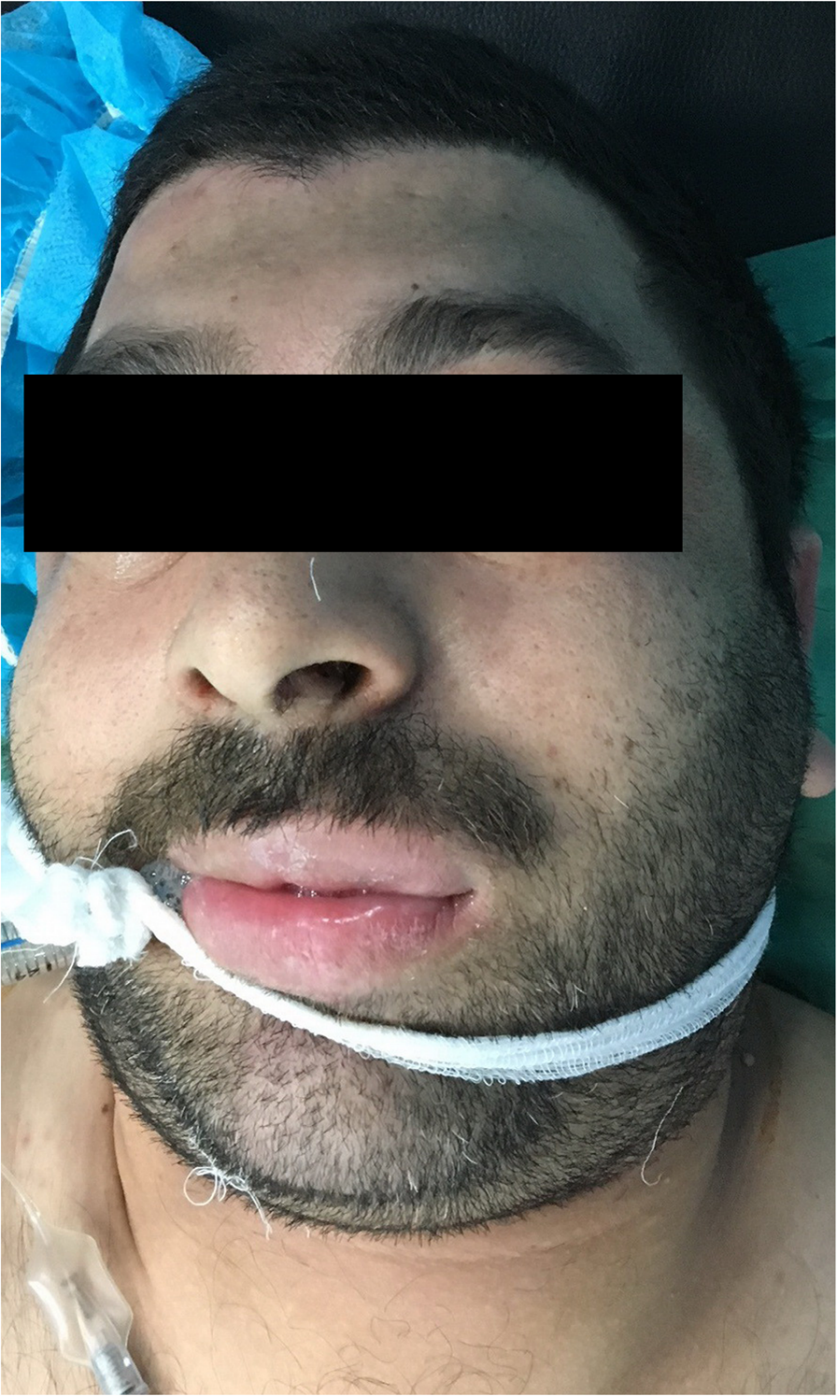
severe swelling of the neck and the face including the eyes lids, the checks, and the lips immediately after changing the patient from prone to supine position

At the end of the surgery and turning the patient into supine position, we noticed severe swelling in the neck and the face including the eyes lids, the checks, and the lips, Fig. 1. Direct Laryngoscopy revealed an edematous tongue, floor of the mouth, glottis, and supraglottic areas without rash association. On auscultation, there were no added breath sounds with normal vital signs. The patient was kept in prone position for 305 min.

A diagnosis of drug induced angioedema was made after exclusion of other causes and intravenous dexamethasone 10 mg, diphenhydramine 25 mg and ranitidine 50 mg were given. He was continuously monitored for progression of the edema and continued to have dexamethasone. The patient remained intubated and was transferred to the intensive care unit. The valsartan was suspected to be the precipitating factor for the angioedema and was therefore discontinued.

**Fig. 2:**
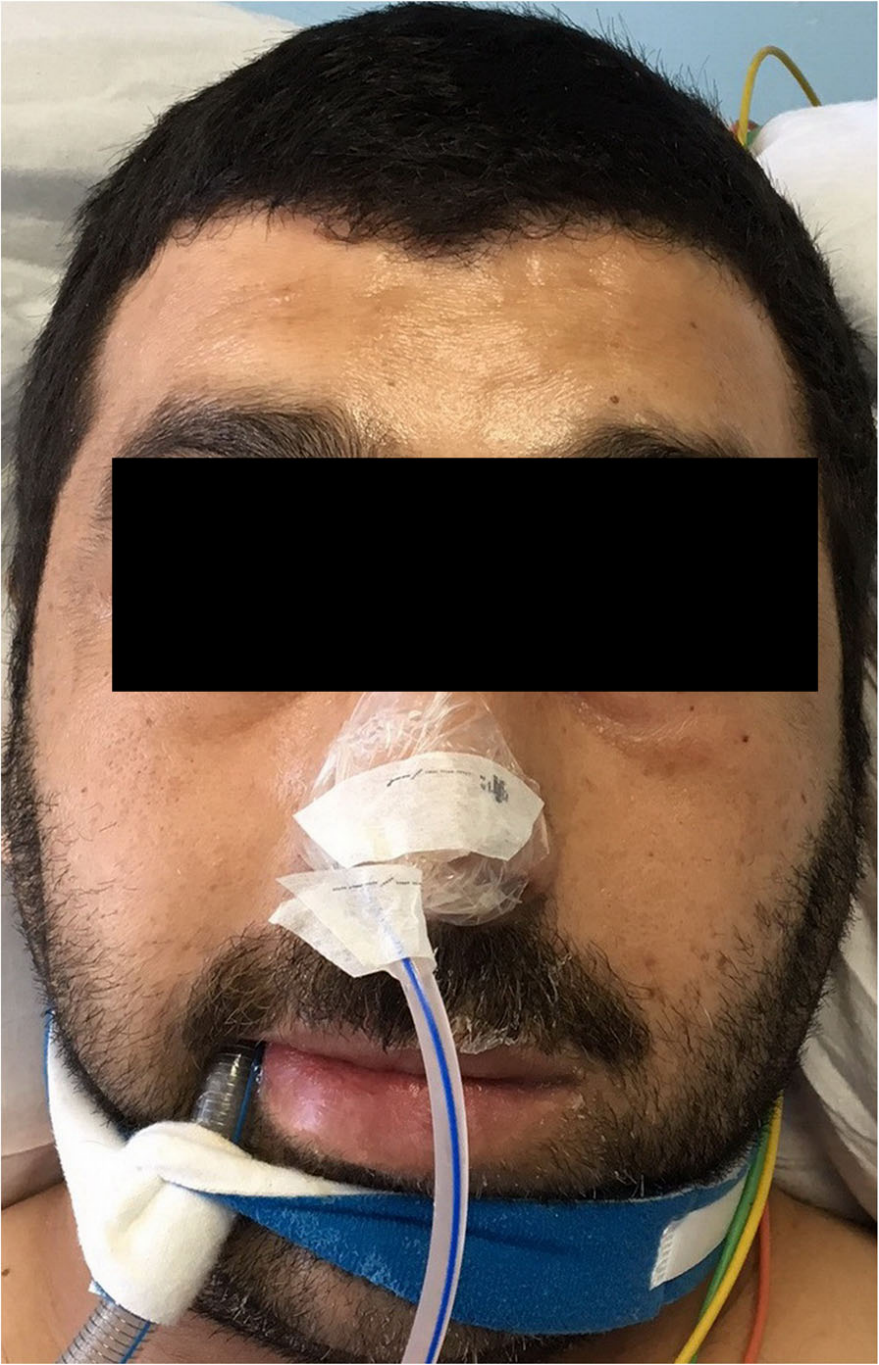
Regression of angioedema after 24 h

**Fig. 3:**
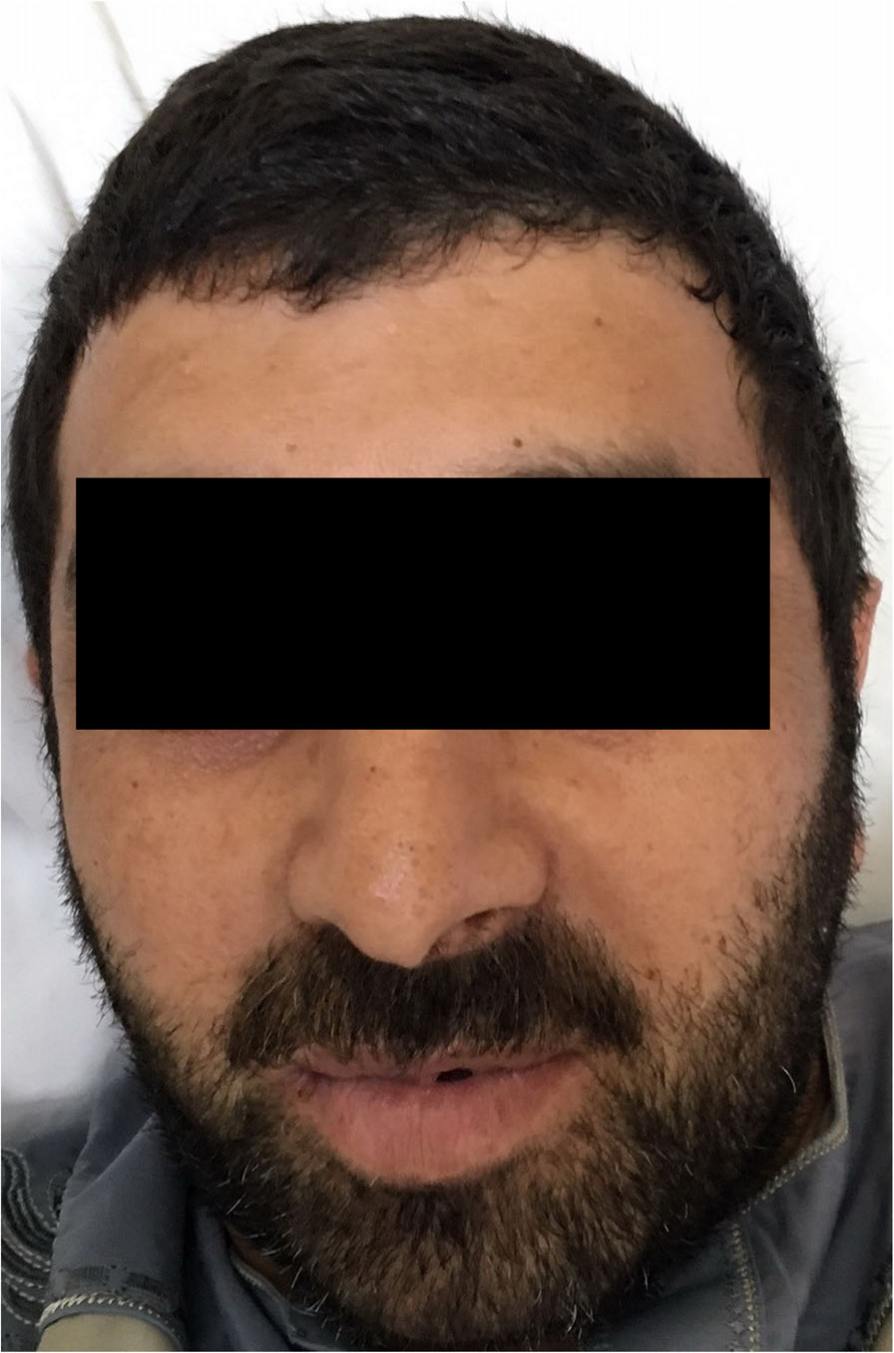
The patient after 72 h with complete resolution of the angioedema

The swelling started to regress after 2 h and significantly within 24 h, Fig. 2. The extubation was done on the second day after a flexible fiberoptic examination revealed normal supraglottic and glottic structures. The facial and neck swelling has resolved completely by the third day, Fig. 3.

The patient was discharged home on the fifth post-operative day without any complications with no history of further attacks of angioedema during his follow up visits in spine clinic.

## Discussion

The differential diagnosis of postoperative face and neck swelling includes; angioedema, allergic reaction and anaphylaxis, and face congestion related to prolonged prone position during surgery [8].

In our case, anaphylaxis was excluded because there were no hypotension, flushing, and other allergic symptoms. The patient had no history of nonsteroidal anti-inflammatory drugs in perioperative period. Furthermore, the only discontinued drug after surgery was valsartan, while cefazolin and morphine were administered daily for three days and he had sustained clinical improvement.

Only three case reports of oropharyngeal swelling during anesthesia in prone position have been reported. One patient underwent suboccipital craniotomy for an Arnold-Chiari malformation. A second case also had Arnold-Chiari malformation and underwent posterior cervical spine decompression. A third case underwent posterior fossa surgery in the prone position.

The proposed mechanism could be excessive head flexion and kinking or obstruction of the internal jugular vein by tracheal tube, which cause obstruction of venous drainage from the lingual and pharyngeal veins.

Anatomical abnormalities of the skull base is a common feature of the three reported cases, which might predispose to venous compression, however this would be tolerated by normal subjects [9].

Shriver et al. [10] reviewed clinical studies reporting complications associated with positioning during lumbar spine surgery. The only reported upper airway complication was unusual intraoperative discovery of a bite injury, producing a cyanotic, edematous, protruding tongue.

In our case, the patient’s head was on head frame in neutral position without neck compression. In addition, the tongue was edematous, pinkish in color after surgery.

Furthermore, there is no reported case of edema in the face, neck, and upper airway after lumber spine surgery; however, there are few reports of such complication in perioperative period in patients on ACE inhibitors, which made us to think of high possibility of drug induced angioedema.

There are different types of angioedema: (a) Mast cell-mediated angioedema where the presence of urticaria, pruritus, cutaneous flushing, as well as history of unexplained hypotension, and near-syncope should be noted, however, these systemic manifestations were not seen in our case, (b) Kinin-related angioedema which usually related to drugs that work on the angiotensin system (eg, ACE inhibitors) with no urticaria or other systemic manifestations, (c) hereditary angioedema which is a rare autosomal dominant disease caused by C1 esterase inhibitor enzyme deficiency and is characterized by multiple angioedema attacks. Our patient has normal C1 esterase level, (d) angioedema of unknown etiologies which are less frequent and represent a chronic condition where patients have repeated attacks of angioedema typically associated with urticaria and other autoimmune conditions [11]. Our patient was healthy with no history of urticaria, and/or previous attacks of angioedema.

ACE inhibitors are the most common cause of drug induced angioedema representing 25% to 39% of cases [8]. Patients taking ACE inhibitors have been reported to have a risk of developing angioedema weeks or years in the course of therapy ranging from 0.1 to 0.5% [3], and it is thought that the prevalence of ACE inhibitor-related angioedema is frequently underestimated, particularly when its presentation is delayed following long-term therapy [11].

Several factors may trigger development of angioedema in patients receiving ACE inhibitors including; previous angioedema, age above 65, nonsteroidal anti-inflammatory drugs, female sex, smoking, seasonal allergies, underlying C1 inhibitor deficiency or dysfunction, history of ACE inhibitor-induced cough, and surgery [12].

The mechanism of ACE inhibitor-induced angioedema is not well known. It is thought that ACE inhibitors inhibit the degradation of bradykinin leading to increase its level in the serum that results in vasodilation and increased vascular permeability especially in the lax tissues of the face [13, 14]. Bradykinin is increased in all patients taking ACE inhibitors; however, only small percentage develops such edema. Therefore, it is likely that factors other than impaired bradykinin degradation are involved in the development of angioedema.

Recent case reports and reviews indicate that patients receiving angiotensin II receptor blockers therapy can also develop similar angioedema. Valsartan has been found to be the most prevalent angiotensin II receptor blocker-induced angioedema [2]. Ten percent of patients with previous ACE inhibitor-induced angioedema develop this adverse reaction after changing the medication to angiotensin II receptor blockers [15].

Elevated bradykinin theory also has been proposed for angiotensin II receptor blockers. Angiotensin II receptor blockers may cause angioedema by inhibition of angiotensin II type 1 (AT1) receptors, leading to increased level of angiotensin II and therefore increase type 2 (AT2) receptor activity that causes increased bradykinin level [12]. However, there is a report that angiotensin II receptor blockers treatment (50 mg losartan) directly increases the serum bradykinin [3].

Angiotensin II receptor blocker-induced angioedema is a diagnosis of exclusion with no specific investigation. We believe that our patient had a severe life threatening angioedema with high probability that the etiology was directly related to the previous treatment with valsartan.

Regardless of the cause, the initial management of angioedema consists of securing the airway and discontinuation any suspected triggering drug. Although angioedema is self-limiting and the swellings will normally subside in approximately 72 h with or without treatment, intravenous corticosteroids and antihistamines are still widely used [16].

Chiu et al. [13] presented a classification system for angioedema to predict airway risk and patients were classified as class 1 when the edema involves the face and oral cavity, class 2 when the edema extends to floor of the mouth and oropharynx, and class 3 when glottis and supraglottis involvement occurs.

They recommend that patients with class 2 or 3 required securing their airway by tracheal intubation. According to this classification our patient had class 3 (swelling of the face, tongue, floor of the mouth, and glottis and supraglottis).

## Conclusion

Cases of angiotensin II receptor blocker-induced angioedema have been reported in literature. The precise mechanism of angiotensin II receptor blocker-induced angioedema is still unknown and should be thoroughly investigated. We report a unique case of intraoperative angiotensin II receptor blocker-induced angioedema. Anesthesiologists should be aware of such complication that can happen in patients using angiotensin II receptor blockers.

## Abbreviations

ACE: Angiotensin converting enzyme
AT1: Angiotensin II type 1
AT2: Angiotensin II type 2
